# Genetic diversity and molecular typing of *Listeria monocytogenes * in China

**DOI:** 10.1186/1471-2180-12-119

**Published:** 2012-06-22

**Authors:** Yan Wang, Ailan Zhao, Renfa Zhu, Ruiting Lan, Dong Jin, Zhigang Cui, Yonglu Wang, Zhenchuan Li, Yiting Wang, Jianguo Xu, Changyun Ye

**Affiliations:** 1 State Key Laboratory for Infectious Disease Prevention and Control, National Institute for Communicable Disease Control and Prevention, China CDC, Changbai Road 155, Changping, Beijing, China; 2 Foshan Municipal Bureau of Agriculture, Guangdong Province, China; 3 School of Biotechnology and Biomolecular Sciences, University of New South Wales, Sydney, NSW, 2052, Australia; 4 Maanshan Center for Disease Control and Prevention, Anhui Province, China

## Abstract

**Background:**

*Listeria monocytogenes * can cause invasive diseases in humans and farm animals and is frequently isolated from dairy products and poultry. Listeriosis is uncommon in China but *L. monocytogenes * has been isolated from foods and food processing environments in China. However little is known of genetic diversity of Chinese *L. monocytogenes * isolates and their relationships with global isolates.

**Results:**

Two hundred and twelve isolates of *L. monocytogenes * from food sources from 12 provinces/cities in China were analysed by serotyping, Pulsed Field Gel Electrophoresis (PFGE) and Multi-locus Sequence Typing (MLST). The predominant serotypes are 1/2a, 1/2b and 1/2c accounting for 90.1% of the isolates. PFGE divided the isolates into 61 pulse types (PTs). Twenty nine PTs were represented by more than one isolates with PT GX6A16.0004 containing the most number of isolates. MLST differentiated the isolates into 36 STs, among which 15 were novel. The 3 most common STs were ST9 (29.1%), ST8 (10.7%) and ST87 (9.2%), accounting for 49.0% of the isolates.

**Conclusions:**

STs prevalent in other parts of the world are also prevalent in China including 7 STs (ST1-ST3, ST5, ST6, ST8, ST9) which caused maternal fetal infections or outbreaks, suggesting that these STs potentially can also cause severe human infections or outbreaks in China. Surveillance of these STs will provide important information for prevention of listeriosis. This study also enhances our understanding of genetic diversity of *L. monocytogenes * in China.

## Background

*Listeria monocytogenes *, a food borne pathogen, is frequently isolated from dairy products and poultry. It can cause invasive diseases in humans and farm animals, including meningitis, fetal loss, sepsis, and febrile gastroenteritis [1]. Although *L. monocytogenes * is an uncommon human pathogen, it has a disproportionate share of the food borne disease burden. For example, there were only 2,500 illnesses annually in the US but *L. monocytogenes * infections account for 4% of all hospitalizations and 28% of all deaths from food borne diseases [2]. A large outbreak occurred in the Maritime Provinces of Canada in 1981, which provided the first evidence for transmission of listeriosis by food-borne *L. monocytogenes *[3,4]. Since then, many outbreaks of listeriosis have been reported: six in the US include two in Massachusetts in 1983 and 2007 [5,6], one in California in 1985 [7], one in Illinois in 1994 [8], a multi-states outbreak in 2002 [9] and one most recent outbreak in 2011 [10]; one in Canada in 2008 [11] and five in Europe including one each in France in 1992 [12], Switzerland between 1983 and 1987 [13], Sweden in 1995 [14], Italy in 1997 [15] and Finland in 1999 [16].

*L. monocytogenes * is a diverse species and has been typed using a range of subtyping procedures to examine the epidemiology and population genetics. Serotyping is a classic subtyping method with limited discriminatory power. Thirteen serotypes of *L. monocytogenes * are recognized. Three serotypes (serotype 1/2a, 1/2b and 4b) cause the majority of clinical cases and serotype 4b causes the majority of human epidemics [17]. Pulsed Field Gel Electrophoresis (PFGE) provides higher discrimination than serotyping and is often considered the standard subtyping method for source tracking and epidemiologic investigations [18]. Multi-locus sequence typing (MLST) based on nucleotide sequences of housekeeping genes has also been shown to be highly discriminatory for *L. monocytogenes *[19], with an added advantage that it provides unambiguous results comparable among laboratories via the internet. *L. monocytogenes * is well recognized to be divided into 3 lineages [20,21]. In a recent study, Wiedmann *et al.* discovered a fourth lineages, however, lineages III and IV were rare [22]. Brisse *et al.* established a standardized MLST scheme using seven housekeeping genes and used it to characterized a large collection of *L. monocytogenes * isolates [23]. An MLST database was also established which allows other researchers to submit new MLST data and facilitates international comparison although the use of unpublished MLST data in the database is restricted.

Listeriosis is uncommon in China and there was no report of human outbreaks so far. This may be partly due to lack of surveillance of clinical listeriosis. Surveillance of *L. monocytogenes * in foods has been implemented nationally and *L. monocytogenes * has been isolated from foods and food processing environments in China including chicken, pork, fish and vegetables [24-27]. Zhou *et al* analyzed 38 *L. monocytogenes * isolates from food products and sewage samples in China using single gene sequencing of the *actA* gene while Jiang *et al*. [28] characterized 20 *L. monocytogenes * isolates from Zhejiang province of China by a non-standardized MLST scheme based on three virulence genes and four housekeeping genes. Neither of these sequence data allows one to make a comparison with the current extensive international MLST data. In this study, isolates were obtained from different food products through food surveillance from 12 provinces or cities across China, and analyzed by serotyping, PFGE and MLST to further determine the genetic diversity of Chinese *L. monocytogenes * isolates and to compare Chinese isolates with international isolates from published studies.

## Methods

### *L. monocytogenes * isolates

Two hundred and twelve isolates of *L. monocytogenes * from 12 provinces/cities in China were used for this study. The isolates were from different food products isolated by local food surveillance laboratories between 2000 and 2008 (Table 1). Food surveillance was generally conducted with random sampling from open markets and production plants periodically based on national surveillance guidelines. Our isolates were a random sample of these surveillance isolates and were not known to be linked by transmission chain or food sources. The isolates were identified by PCR targeting *hly* fragments specific for *L. monocytogenes * and serotyped using antiserum against somatic and flagella antigens according to the instructions of the manufacture (Denka Seiken, Tokyo, Japan).

**Table 1:** **Summaries of *Listeria monocytogenes * isolates used in this study by sequence types**

**Sequence type**	**No. of isolates**	**Serotype**	**Year**	**City/province**	**Source**
ST1	5	4b	2001, 2005-2006	D, E, F, I	Pork, cooked meat, food*
ST2	5	4b	2001	D, G	Pork, beef, cooked meat, chicken
ST3	8	1/2b	2001-2002, 2005-2006	B, C, D, H, L	Cooked meat, chicken, food*
ST5	5	1/2b	2001, 2005, 2007	C, D, K, L	Cooked meat, pork
ST6	1	4b	2001	D	Chicken
ST7	7	1/2a	2005-2006	H	Food*
ST8	22	1/2a	2001-2002, 2005–2006, 2008	A, B, D, H, I, J	Cooked meat, pork, beef, ice cream, chicken, food*
ST9	60	1/2c	2000-2002, 2004-2008	A, B, C, D, G, H, I, K, L	Pork, chicken, cooked meat, aquatic product, beef, mutton, fish, food*
ST14	3	1/2a	2005, 2006, 2008	H, J, L	Chicken, food*
ST59	5	1/2b	2001, 2005, 2007	I, K, L	Ice cream, pork, chicken
ST83	1	1/2c	2002	B	Pork
ST87	19	1/2b, 3b	2001-2007	B, D, E, G, H, K, L	Pork, vegetables, chicken, cooked meat, mutton, food*
ST101	6	1/2a	2003, 2005-2006	D, L	Pork, chicken, food*
ST120	2	1/2a	2002, 2008	A, B	Pork, meat
ST121	13	1/2a	2001-2002, 2004-2008	A, B, C, D, H, J, K, L	Pork, chicken, cooked meat, food*
ST122	12	1/2c	2000-2001	G	Pork, chicken, cooked meat, vegetables, mutton
ST124	1	1/2a	2005	D	Pork
ST155	7	1/2a, 3a	2001-2002, 2008	A, B	Chicken, mutton
ST199	9	1/2a	2001-2002, 2005-2006	C, D, G, H	Pork, cooked meat, chicken, food*
ST218	1	1/2b	2005	L	Chicken
ST288	1	1/2b	2005	L	Chicken
ST295	1	1/2b	2001	I	Ice cream
ST296	1	1/2b	2001	D	Chicken
ST297	1	1/2a	2003	D	Pork
ST298	1	4b	2006	F	Food*
ST299	2	4c	2006	E, F	Fish, food*
ST300	1	1/2a	2004	B	Chicken
ST301	1	1/2c	2002	B	Beef
ST302	2	1/2a, 3a	2002	B	Chicken
ST304	2	1/2c	2000	G	Beef, pork
ST305	1	1/2b	2001	G	Cooked meat
ST306	1	1/2c	2001	G	Vegetables
ST307	2	1/2a	2003-2004	B, D	Pork
ST308	1	4b	2006	F	Food*
ST310	1	1/2b	2005	E	Meat
ST312	1	1/2c	2000	G	Pork

* Food indicates the isolates from unknown food source.

A: Anhui B: Beijing C: Chongqing D: Fujian E: Guangdong F: Guangxi G: Henan H: Hubei I: Jiangsu J: Shanghai K: Sichuan L: Zhejiang.

### Pulsed-field gel electrophoresis analysis

Agarose plugs were prepared and PFGE was performed, according to Centers for Disease Control and Prevention PulseNet standardized procedure for typing *L. monocytogenes *. The Pulse-Net protocol recommends two restriction enzymes *Apa*I and *Asc*I for PFGE. Only *Asc*I was used in this study since *Asc*I PFGE patterns are more easily analyzed than *Apa*I patterns by the BioNumerics software and operators [29]. Briefly, genomic DNA was prepared by mixing 240 μl of standardized cell suspension and 60 μl of 10 mg/ml lysozyme solution, followed by incubation at 37°C for 10 min. Sample plugs were digested with 25 U of *Asc*I (Takara, Beijing, People’s Republic of China) at 37°C for 3 h. Plugs were then loaded on 1% Seakem Gold agarose gel in 5 × TBE (45 mM Tris, 45 mM Borate, 1 mM EDTA) and electrophoresed on a CHEF DR III apparatus (Bio-Rad, Beijing, People’s Republic of China), using the following parameters: initial switch time, 4 s; final switch time, 40 s; run time, 22 h; angle, 120°; gradient, 6 V/cm; temperature, 14°C; ramping factor, linear. Gels were stained with ethidium bromide and visualized on a UV transilluminator. *Salmonella enterica* serovar Braenderup strain H9812 restricted with *Xba*I was used for molecular weight determinations in all PFGE gels. Similarities between restriction endonuclease digestion profiles were analyzed by using Unweighted Pair Group Method with Arithmetic Mean (UPGMA) of BioNumerics software (Applied Maths, Kortrijk, Belgium).

### Multi-locus sequence typing and phylogenetic analysis

The MLST scheme available at http://www.pasteur.fr/recherche/genopole/PF8/mlst/Lmono.html was used. The nucleotide sequences of internal fragment of the following genes, *acbZ* (ABC transporter), *bglA* (beta-glucosidase), *cat* (catalase), *dapE* (succinyl diaminopimelate desuccinylase), *dat* (D-amino acid aminotransferase), *ldh* (L-lactate dehydrogenase), and *lhkA* (histidine kinase), were obtained by PCR using published primers (Table 1) with the exception of primers for *lhkA*. A new pair of primers for *lhkA* (*lhkA*F 5′-GTTTTCCCAGTCACGACGTTGTATTATCAAAGCAAGTAGATG-3′ and *lhkA*R 5′-TTGTGAGCGGATAACAATTTCTTTCACTTTTTGGAATAATAT-3′) were designed to amplify the *lhkA* gene from the isolates which had no amplification products when the published primers were used. A 50-μl reaction was composed as follows: 5.0 μl of 10 × pfu buffer with 1.5 mM MgCl_2_, 125 μM each of deoxynucleoside triphosphate mix, 0.2 μM forward and reverse primers, 0.5U of pfu DNA polymerase, and 2U of rTaq DNA polymerase. The PCR amplification conditions were as follow: 94°C for 4 min and 30 cycles of 94°C for 30 s, 52°C for 30s, and 72°C for 2 min, followed by one cycle of 72°C for 10 min and hold indefinitely at 4°C. The purified PCR products were sent for sequencing commercially.

For each isolate, the allele combination at the 7 loci defines an allelic profile or sequence type (ST). Minimum spanning tree (MST) analysis was used to infer relationships among the isolates and was done using BioNumerics (Applied Maths, Belgium). Neighbor-joining tree of the seven concatenated housekeeping gene sequences was constructed using MEGA 4.0 [30]. A clonal complex (CC) is defined based on eBURST algorithm with member STs differing by only one of the 7 MLST genes [23].

## Results

### Serotyping

The 212 isolates used in this study were typed into seven of the 13 known serotypes: 1/2a, 1/2b, 1/2c, 3a, 3b, 4b and 4c. The most frequent serotypes are 1/2c, 1/2a and 1/2b with a frequency of 36.8%, 33.5% and 19.8% respectively. The remaining 4 serotypes account for only 9.9% of the total isolates.

### Pulsed-field gel electrophoresis

**Figure 1:**
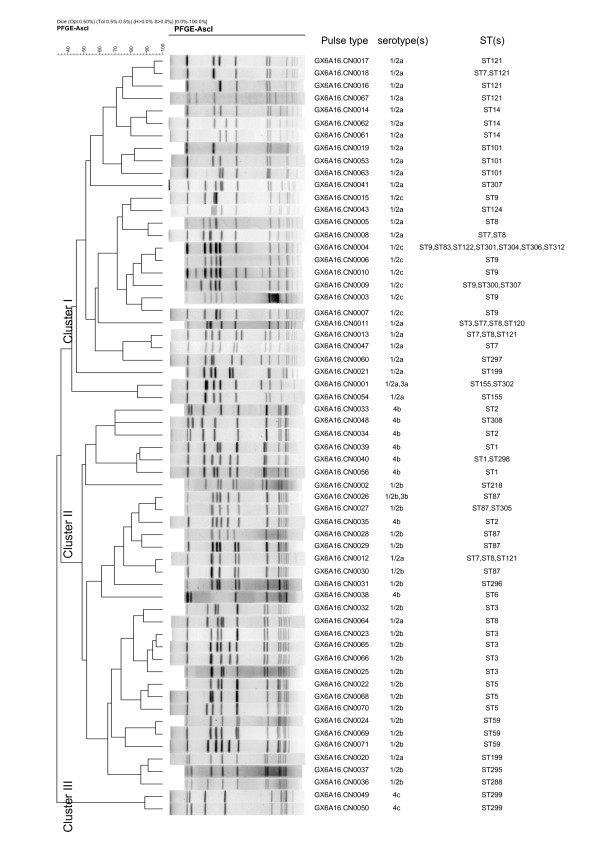
**Relationships of the isolates based on PFGE.** The 212 *L. monocytogenes * isolates from China were analyzed by PFGE using *Asc* I. The dendrogram were constructed using UPGMA. The corresponding pulse type, serotype(s) and ST(s) were shown alongside the dendrogram on the right.

PFGE analysis divided the 212 isolates into 61 pulse types (PTs). PTGX6A16.0004 was predominant and accounts for 26.5% of the isolates, followed by GX6A16.0011 (17 isolates), and GX6A16.0009 (13 isolates). Thirty two PTs (52.5%) were represented by only a single isolate. A UPGMA dendrogram was constructed for the 61 PTs based on presence or absence of bands. The PTs are divided into 3 clusters. Cluster I contained all serotype 1/2c isolates, the majority of serotype 1/2a isolates. Cluster II contained all serotype 4b and 1/2b isolates and the remaining serotype 1/2a isolates. Cluster III contained only two PTs (GX6A16.0049 and GX6A16.0050) belonging to serotype 4c and are very divergent (Figure 1).

### Multi-locus sequence typing

The 212 isolates were divided into 36 sequence types (STs), among which 21 STs have previously reported in other countries, 15 STs (ST295-ST302, ST304-ST308, ST310 and ST312) were novel. The most common STs are ST9 (29.1%), all of which are serotype 1/2c, ST8 (11.7%) with all isolates belonging to 1/2a, and ST87 (10.7%) with all except one being 1/2b isolates and the exception being a 3b isolate. Fifteen STs (41.7%) were represented by only one isolate (Table 1).

**Figure 2:**
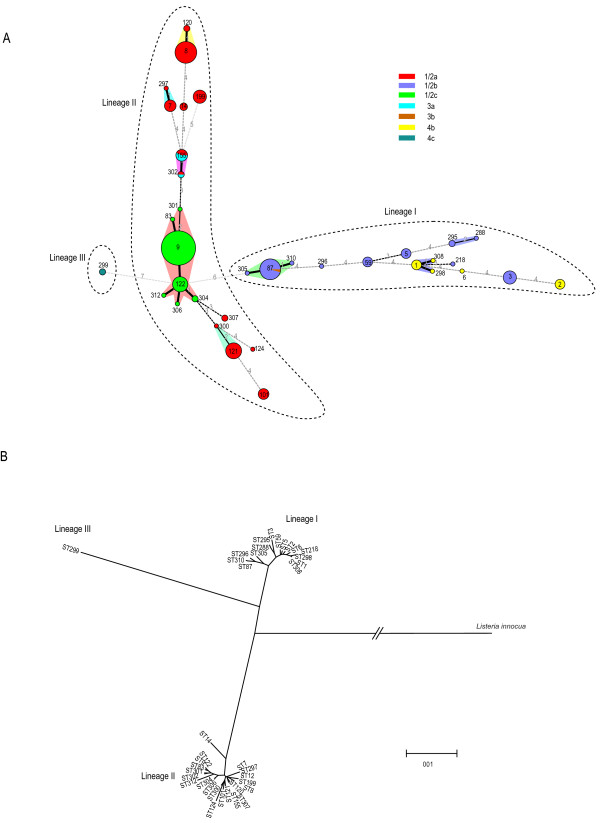
**Genetic relationships of the isolates based on MLST. A**) The minimum spanning tree of the 36 STs from China. Each circle corresponds to a sequence type. The shadow zones in different color correspond to different clonal complexes. The size of the circle is proportional to the number of the isolates, and the color within the cycles represents the serotypes of the isolates. **B**) Neighbor-joining tree of *L. monocytogenes * sequence types constructed using the concatenate sequences of seven housekeeping genes. *Listeria innocua* was used as an outgroup. Lineages are marked on both trees which were shown using dotted boundary lines in **A**.

The 36 STs were grouped into six clonal complexes and 18 singletons according to eBURST algorithm (Figure 2A). They were divided into three lineages as defined by Wiedmann [20]. Lineage I includes two clonal complexes: CC1 and CC87, of serotypes1/2b, 3b and 4b, and nine singletons of which seven are serotype 1/2b and two are serotype 4b. Lineage II includes four clonal complexes: CC7, CC8, CC9 and CC155. CC9 contains the largest number of STs including ST9, ST83, ST122, ST304, ST306 and ST312. All isolates in CC9 were serotype 1/2c. CC7 and CC8 were serotype 1/2a while CC155 includes both serotypes 1/2a and 3a. The singletons in this lineage were all serotype 1/2a, except for one isolate being serotype 1/2c (ST301). Lineage III contained two isolates, both belonging to ST299 and serotype 4c.

## Discussion

### Correlation among serotype, pulse type and sequence type

In most cases, *L. monocytogenes * isolates of the same PT and ST belong to the same serotype but there were exceptions. Two isolates (LM 078 and LM 099) of the same PT (GX6A16.0026) and ST (ST87) are different serotypes (3b and 1/2b respectively). Among the five isolates of pulse type GX6A16.0001 and ST155, four and one were serotype 3a and serotype 1/2a respectively. The observation indicates that serotype 3a and 1/2a can be easily switched. Additionally there were 13 cases of the same PT but different STs. For example, of 58 isolates (all serotype 1/2c) with PT GX6A16.0004, 40 isolates were ST9, 12 isolates were ST122, 2 isolates were ST304, and one each was ST83, ST301, ST306 and ST312 respectively. These STs were all grouped into CC9 except for ST301, which shares 5 of the 7 alleles with ST9. In another case, of the 13 isolates of PT GX6A16.0009, 11 were ST9, one each was ST300 and ST307, both of which shared only 3 alleles with ST9. The Simpson’s diversity index for PFGE is 0.913 which is only slightly higher than that of MLST (0.891). However the discriminatory power for PFGE can be increased by using an additional enzyme *Apa*I as recommended by the PulseNet protocol [31] and our study affirms the need to use the additional enzyme for outbreak investigations as discriminatory power of *Asc*I is low.

### Comparison of isolates from China with international isolates

**Figure 3:**
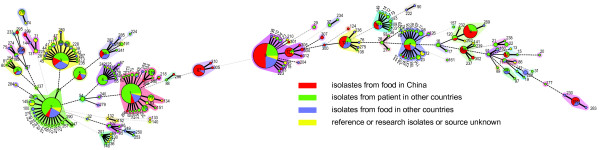
**Genetic relationship of the 212 Chinese isolates and 657 global isolates.** A minimum spanning tree was constructed based on 36 STs (212 isolates) from this study and 196 STs (657 isolates) from the studies of Ragon *et al*. and Chenal-Francisque *et al*. The size of the circle is proportional to the number of the isolates, and the sources of the isolates were colored as shown in figure.

The STs from this study were compared with 196 STs from an analysis of 657 global isolates from the study of Rogon *et al*. [23] and Chenal-Francisque *et al*[32], we found that 16 of the 36 STs in China shared the same sequence types with isolates from patients in other countries, including maternal-fetal infections, central nervous system infections and bacteriemia patients (Figure 3). Seven STs containing nearly half or more than half of the isolates from Rogon *et al*. [23] including ST1 (26/44 isolates), ST2 (10/24), ST3 (10/25), ST5 (15/19), ST6 (6/7), ST8 (5/9) and ST9 (13/28) caused maternal-fetal infections. In addition, at least 2 of these STs have caused outbreaks in Europe. ST1 caused outbreaks in France in 1989 and in Sweden in 1995 while ST2 caused an outbreak in Italy in 1997. These same sequence types isolated from food sources and in particular ST8 and ST9 were the 2 most common STs in China. Based on these observations, we conclude that these STs have the potential to cause disease in humans in China. Human listeriosis has been rarely reported in China which may be contributed by poor disease awareness, lack of diagnostic tools and lack of surveillance.

This study also affirms the recent report by Chenal-Francisque *et al.*[32] that some clones including epidemic clones are prevalent worldwide and globally distributed. In that study, however, there are only 5 isolates from China to represent Eastern Asia. Our study adds a broader picture from China to the global clones and substantial genetic diversity of *L. monocytogenes * to the global gene pool from China. The 15 novel STs from this study were not found in the study of Chenal-Francisque *et al.*[32], although 9 novel STs fall into their clonal complexes. Further, prevalent STs in China may be rare elsewhere, for example, the third most prevalent ST, ST87 was seen with a single isolate from Colombia in the global set of Chenal-Francisque *et al.*[32].

Our isolates were from over nine food types and only those from chicken and pork had sufficient numbers for comparison of clonal diversity between food types. There were 48 samples each from chicken and pork. In both food types, ST9 was predominant with 11 and 30 isolates in chicken and pork respectively. Genetic diversity is higher from chicken samples as measured by Simpson’s index of diversity with 0.906 and 0.722 for chicken and pork respectively.

### Population structure and recombination of *L. monocytogenes *

Many studies have shown that *L. monocytogenes * can be divided into three lineages [20,21]. Lineage I includes isolates of serotypes 4b, 1/2b, 3b, 4d and 4e, containing all food-borne-epidemic isolates as well as isolates from sporadic cases in humans and animals. Lineage II includes isolates of serotypes 1/2a, 1/2c, 3a and 3c, containing both human and animal isolates, but is seldom associated with food-borne epidemics and predominantly isolated from food products. Lineage III are mostly serotypes 4a and 4c and is predominantly isolated from animals [20,33]. All our isolates can be allocated into one of the three lineages. The majority of our isolates (154 out of 212, 72.6%) including the 60 isolates of ST9 (the most frequent ST in China) belonged to lineage II since our isolates were from food sources. Fifty six isolates (26.4%) belonged to lineage I while only two isolates, both being ST299 belonged to lineage III.

We used the counting method used by Feil *et al*. [34] to determine the ratio of recombination to mutation per locus. A single allelic difference between STs within a clonal complex was attributed to either mutation if the difference was a single base or recombination otherwise. We found that alleles are three times more likely to change by mutation than by recombination (r/m = 0.306). This estimate is similar to that (r/m = 0.197) reported by Ragon *et al. *[23]. Interestingly, five of the eleven recombination events observed were in the same gene (*abcZ*), three in CC9, one in CC87 and one in CC155. A possible explanation for the high frequency of recombination in *abcZ* is positive selection. However Ragon et al. [23] showed that the ratio of non-synonymous/synonymous substitution rate (Ka/Ks) of *abcZ* was 0.014 suggesting that *abcZ* was not under positive selection. An alternative explanation is that *abcZ* is linked to a nearby gene that is under positive selection and has undergone recombination by hitch-hiking. This scenario has been observed to have occurred in genes around the O antigen encoding locus in *E. coli* and other species [26]. Examination of sequences 30 kb up and down stream of *abcZ* based on the genome sequence of isolate EGD-e did not identify a gene or gene cluster that is likely to be under positive selection.

## Conclusions

This study analyzed 212 isolates from food sources in China by serotyping, PFGE and MLST, and showed that the common STs in China are also the prevalent STs in other countries, many of which contain isolates from human infections. A corollary of this observation is that the STs that have caused outbreaks of human infections in other parts of the world have the potential to cause outbreaks in China. However, there is hardly any data on human *L. monocytogenes * infections in China partly due to the lack of clinical listeriosis surveillance. A recent report of 6 cases of neonatal listeriosis in a Beijing hospital of 13,372 live births in 2008 highlights that the disease may be more common in China [35]. With the country becoming more effluent, food distribution, storage and consumption patterns have also changed. Since the isolates from food sources as shown in this study clearly have the potential to cause disease, there is a need for surveillance of clinical listeriosis and implementation of prevention strategies to prevent emergence and outbreaks of human *L. monocytogenes * infections in China. The findings also have implications for other countries where there is no surveillance system for *L. monocytogenes *.

## Authors’ contributions

YW performed the serotyping and MLST typing work and drafted the manuscript. AZ performed strain identification. RZ, DJ and ZL performed the PFGE experiments. ZC and YW participated in the analysis of PFGE data. RL participated in data analysis and revised the manuscript. YW collected some strains. JX involved in project design. CY managed the project and co-wrote the manuscript. All authors read and approved the final manuscript.
